# Inflammaging and Immunosenescence in the Post‐COVID Era: Small Molecules, Big Challenges

**DOI:** 10.1002/cmdc.202400672

**Published:** 2024-12-16

**Authors:** Fabio Francavilla, Francesca Intranuovo, Gabriella La Spada, Enza Lacivita, Marco Catto, Elisabetta Anna Graps, Cosimo Damiano Altomare

**Affiliations:** ^1^ Department of Pharmacy-Pharmaceutical Sciences University of Bari Aldo Moro Via E. Orabona 4 70125 Bari Italy; ^2^ ARESS Puglia - Agenzia Regionale strategica per la Salute ed il Sociale Lungomare Nazario Sauro 33 70121 Bari Italy

**Keywords:** Endocannabinoid system, Formyl peptide 2 receptor, Immunosenescence, Inflammaging, Long-COVID, Monoamine oxidases, Oxidative stress

## Abstract

Aging naturally involves a decline in biological functions, often triggering a disequilibrium of physiological processes. A common outcome is the altered response exerted by the immune system to counteract infections, known as immunosenescence, which has been recognized as a primary cause, among others, of the so‐called long‐COVID syndrome. Moreover, the uncontrolled immunoreaction leads to a state of subacute, chronic inflammatory state known as inflammaging, responsible in turn for the chronicization of concomitant pathologies in a self‐sustaining process. Anti‐inflammatory and immunosuppressant drugs are the current choice for the therapy of inflammaging in post‐COVID complications, with contrasting results. The increasing knowledge of the biochemical pathways of inflammaging led to disclose new small molecules‐based therapies directed toward different biological targets involved in inflammation, immunological response, and oxidative stress. Herein, paying particular attention to recent clinical data and preclinical literature, we focus on the role of endocannabinoid system in inflammaging, and the promising therapeutic option represented by the CB2R agonists, the role of novel ligands of the formyl peptide receptor 2 and ultimately the potential of newly discovered monoamine oxidase (MAO) inhibitors with neuroprotective activity in the treatment of immunosenescence.

## Graphical abstract

The post‐COVID syndromes are strictly dependent from inflammaging and immunosenescence, for which the available treatments, particularly immunotherapy, have limited impact and safety issues. Alternative targets are under validation to disclose, besides anti‐inflammatory drugs, new small molecular entities able to counteract the imbalance of inflammatory response in aged patients.

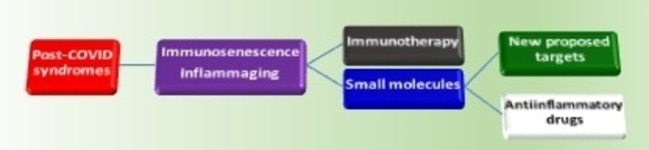

## 1 Introduction

Aging is a phase of biological development, governed by various physical and biological mechanisms, in which a gradual decline of the organism is observed.^[1]^ From a medical‐scientific point of view, aging is a degeneration process that involves cells, tissues and organs and leads to old age (as the last phase of the life cycle). In this period of progressive physical decay, there is also a slowdown and alteration of the normal physiological functions of the organism. Aging is a very complex multifactorial process whose characterization depends on the integration of several interplaying systems.[^2^, ^3^] For these reasons it is difficult to suitably define the complexity of this process.

Certainly, with the increase of age, the immune system undergoes a series of changes and disorders, a phenomenon termed as immunosenescence. Since the early 1900s, the study of aging and age‐related diseases (ARDs) has highlighted innumerable progresses in the understanding of the physiopathological alterations of the immune system, which appear to characterize the specific adaptive immune response.[^4^, ^5^, ^6^, ^7^, ^8^, ^9^] Relevant changes have also been highlighted in the innate immune response during immunosenescence.[^10^, ^11^] The crucial change in immunosenescence of the adaptive component of the immune response concerns the histological changes of the thymus, a progressive process that begins around the age of 20 and runs throughout the life of the individual (approximately 70 years).[^12^, ^13^] In a broad sense, the immune tissue of the thymus shrinks, leaving room for fatty tissue and fibrotic scar tissue. From a cellular and functional point of view, this leads to a reduction in the production of CD4+ and CD8+ T cells that have never encountered the related antigen, with a consequent increase in memory T lymphocytes,[^14^, ^15^] which lack the ability to recognize a new antigen.^[16]^ This imbalance reduces the defensive immune response of the elderly who are more prone to infectious diseases and cancer.[^17^, ^18^] However, the ability of memory T cells to produce T lymphocytes independently of the thymus,^[19]^ as well as concerns about the age‐related decrease in native T lymphocytes,^[20]^ have led to the search for new ways to study immunosenescence. Exposure to stress induces two types of immune response: controlled and adequate or prolonged and excessive which can become chronic.^[21]^ Memory T lymphocytes, produced in this process, become senescent and unable to function properly, resulting in a pro‐inflammatory state of production of reactive cytochemicals and mediators.^[22]^ With aging, the pool of proinflammatory mediators drives T lymphocytes to malfunction and trigger a series of cellular changes,^[23]^ such as stiffening of cell membranes, reduction of the normal transit of mediators between cells, and impairment of the signaling pathways, triggering the immune response to inflammation.[^24^, ^25^] What has been described above falls within the features of normal aging defined as physiological at the basis of ARDs but could also explain pathological aging.

Immunosenescence also affects cells of innate immunity (monocytes, macrophages, NK cells, neutrophils, and others) that can respond differently to different stimuli.[^26^, ^27^] The main functions of innate immunity include: (i) physical and chemical barrier effect to block and/or reduce the progression of internal and external damage; (ii) recruitment of immune cells at the sites of infection through the production of chemical factors; (iii) complement activation to clear cellular debris and antigen‐antibody complexes; (iv) activation of the adaptive immune system by the mechanism of antigen presentation.

With aging, phagocytic cell functions (phagocytosis, cytokine production, and antigen presentation) are impaired.[^21^, ^28^] The phagocytic cells of the elderly are always activated and constantly produce proinflammatory mediators (cytokines and reactive oxygen and nitrogen species),^[29]^ ultimately causing damage by overregulation.^[20]^ From all these evidences it has been established that for the two components of the adaptive and innate immune system there is a constant and latent increase in the proinflammatory tone leading to inflammation.^[20]^ Prolonged exposure to stress challenges the normal physiological defense mechanisms by activating an adaptive immune response.^[30]^

The above‐described alteration of the immune system triggers a chronic inflammation process,[^31^, ^32^, ^33^] for which a first attempt to find a common denominator for all its signs and symptoms was made by Franceschi et al. in 2000, who first introduced the concept of inflammaging.^[34]^ Inflammaging is a sterile, non‐resolving, low‐grade and chronic inflammation that progressively increases with age.^[35]^ When kept below a threshold value, inflammation triggers the sophisticated adaptive immune response that is not always harmful to the body, together with an anti‐inflammatory response that counteracts the damage. In contrast, immunosenescence is linked to the onset of chronic inflammatory diseases in the elderly.^[36]^

Aging is not a disease by itself, but it makes the organism more sensitive to neurodegenerative diseases (Alzheimer's disease (AD), Parkinson's disease (PD), senile dementia, depression),[^37^, ^38^, ^39^] musculoskeletal system damage (rheumatoid arthritis, sarcopenia, osteoporosis, osteoarthritis),[^40^, ^41^, ^42^] cancer (carcinoma, melanoma),[^43^, ^44^] cardiovascular diseases (atherosclerosis, hypertension),[^32^, ^45^] metabolic diseases (type 2 diabetes, obesity, metabolic syndrome).[^46^, ^47^] For many of the above pathologies, inflammaging can be a cause and, in turn, a consequence as well of the altered physiological framework, thus contributing to a self‐sustaining loop. The same interplay occurs in the case of the so‐called long‐COVID syndrome, characterized by a weakened immune response to infections and a state of chronic illness. While the presence of an altered immune response and a debilitating state of inflammaging makes the organism more exposed to COVID,^[48]^ the infection mediates in turn the immune perturbations (T‐cell receptor signaling, regulatory T‐cell function) and the dysregulation of inflammatory mediators (reactive oxygen species (ROS), cytokine network) observed in long‐COVID of aged patients.^[49]^

The concept of age‐related inflammation plays a crucial role in the aging process. Inflammaging and immunosenescence can be seen as the sum of all the reactions that take place in the body.^[34]^ The whole trend can be adaptive and allows a slow and positive aging or, if the stress level reaches a certain threshold of the pro‐inflammatory tipping point, it leads to premature, pathological, and dysfunctional aging. Inflammaging can therefore be harmful or adaptive, depending not only on stress conditions but also on the genetics of the individual and on the surrounding environment.^[50]^

Over the years, new biological players in insurgence and progression of chronic inflammation have been highlighted, widening the choice of therapeutic alternatives. According to the definition of inflammaging, it could be interesting to understand whether it can be faced with anti‐inflammatory, pro‐resolving and antioxidant compounds to counteract chronic inflammation. In this context, addressing inflammaging by modulating cellular senescence may result crucial, and this objective may be achievable with drugs referred to as senotherapeutics, categorized as senolytics and senomorphics.^[51]^

This review aims at exploring the role of some targets, that have not yet been fully exploited, and related ligands for their therapeutic potential in the treatment of inflammaging. In a few cases, we highlight a possible multitarget activity as improving feature for the treatment of such a multifaceted disease, lying on the involvement of such targets in complex, often mutually influencing, metabolic pathways.

## 2 Small Molecules for Long‐COVID Syndrome: Clinical Evidence

**Figure 1:**
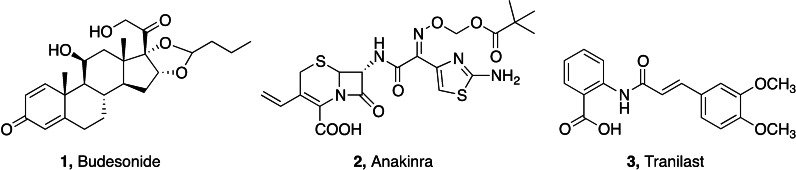
Proposed drugs for the treatment of long‐COVID syndrome.

The SARS‐CoV‐2 pandemic has not only emerged as devastating life‐threatening infection, but also for its long‐term sub‐acute complications. Clinical evidence indicated anti‐inflammatory drugs and immunosuppressors as effective treatments of COVID‐related inflammaging.^[52]^ Dexamethasone has been successfully used in the management of both acute and chronic phases of SARS‐CoV‐2 infection, particularly in patients requiring oxygen hyperventilation.^[53]^ Another glucocorticoid drug, budesonide **1** (Figure 1), modulated inflammatory response in patients hospitalized at the early stage of COVID‐19, leading to a limitation of epithelial damage in respiratory system.^[54]^ Anakinra **2** (Figure 1), a small molecular immunosuppressor acting as inhibitor of receptor antagonist of interleukin 1 (IL‐1), has been suggested as adjutant in severe forms of clinical COVID,^[55]^ although successive clinical trials evidenced contrasting results in long‐term COVID.^[56]^ Tranilast **3** (Figure 1), a modulator of NOD‐like receptor protein 3 (NLRP3) inflammasome used in the management of several inflammatory diseases, has been used in acute COVID patients as adjutant of antiviral therapy, with improved recovery times and milder acute symptoms.^[57]^ Other small molecules of natural origin are supposed to play a role in counteracting inflammaging‐related processes. Among them, melatonin and its metabolites exert several actions as neuroprotective, antioxidant, anti‐excitotoxic agents, all of these features having been deduced from clinical studies and demonstrated in animal models of inflammation and neurodegeneration.^[58]^

## 3 The Role of Microglia in Neuroinflammation

**Figure 2:**
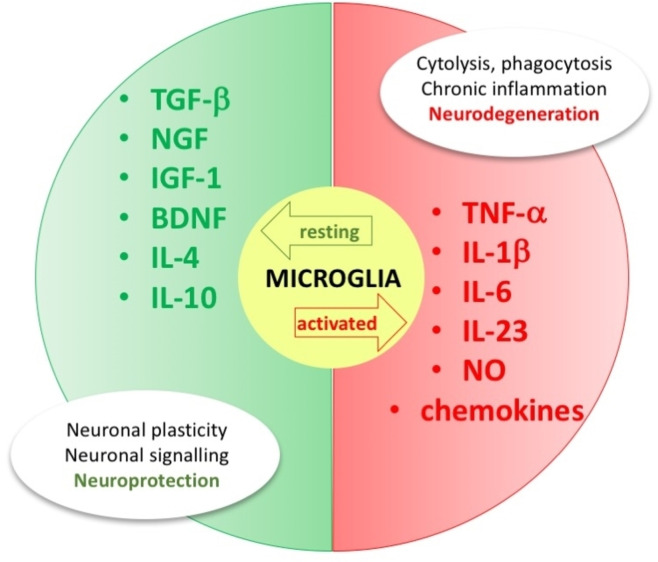
Effects of different phenotypic states of microglia (abbreviations in the text).

Cellular senescence, brain tissue damage, dysfunction in the correct immune response to antigen exposure, high nuclear factor kappa B (NF‐kB) activity, cell aggregation, reactive oxygen species, autophagy, apoptosis, cell damage, epigenetics and genetics, are all events that induce a massive and uncontrolled production of pro‐inflammatory chemical mediators at the brain level, which activate macrophages and microglia. The latter term identifies the resident population of macrophages in the brain. Microglial cells are connected to macrophages from a morphological and phenotypic point of view and are located in the spinal cord, retina and brain.[^59^, ^60^] They play an important role in maintaining the correct functionality of brain circuits throughout life.[^61^, ^62^, ^63^] Microglia is essential, for example, in neuronal plasticity, defined as the brain ability to modify its structure, function and connections and adapt to the stimuli to which it is subjected.[^64^, ^65^] Among the different functions of these sentinels, there are synaptic regulation, neuronal development and regulation, modulation of synaptic transmission.[^66^, ^67^, ^68^] Like macrophages, depending on the stimulus, they can assume different phenotypic states, i. e., resting and activated (Figure 2).^[60]^ The M0 resting state is a quiescent phenotype typical of physiological conditions. In this state, the microglia cells supervise and regulate the entire nervous tissue, like a sentinel, whereas, at the same time, release neurotrophic mediators such as nerve growth factor (NGF), transforming growth factor beta (TGF‐β), insulin‐like growth factor 1 (IGF‐1) and brain‐derived neurotrophic factor (BDNF). Activated microglia cells are involved in several inflammatory‐based neurodegenerative pathologies in two distinct phenotypes, i. e., M1 and M2. The M1 phenotype is the proinflammatory phenotype produced by the presence of dangerous proinflammatory stimuli (e. g. interferon (IFN), lipopolysaccharide (LPS), toll‐like receptor 4 (TLR4) activation) and is associated to the production and release of proinflammatory cytokines, including tumor necrosis factor α (TNF‐α), IL‐6, IL‐23, IL‐1β, IL‐12, chemokines, and nitric oxide (NO).^[69]^ In this state, microglia also achieves cytolytic, phagocytic, and antigen‐presenting capabilities. However, the activation process appears to have multiple phases in which the resting microglia reach a fully activated state by passing through a first reactive and, only subsequently, primed phenotype.[^59^, ^70^]

M2 is the alternative activated anti‐inflammatory phenotype essential to repair tissues and restore homeostasis by preventing cellular necrosis due to chronic inflammation. M2 microglia act by removing cellular debris and promoting the reconstruction of the extracellular matrix. The microglia in this state also produce and release growth factors (TGF‐β, IGF‐1), anti‐inflammatory cytokines (IL‐4, IL‐10, IL‐12), arginase‐1 (catalyzing the production of l‐ornithine which in turn stimulates tissue repair).^[71]^ Due to all these functions, these cells represent the first line of attack of the immune response in the central nervous system (CNS). After identifying neuronal damage or injury, M2 microglia cells move to the affected site to exert their action and remove the dangerous stimulus. When the immune response is triggered, the repair processes are initiated, and homeostasis is then restored. If all this does not happen, the microglia will sustain an inflammatory response over time, which ultimately produces neurotoxicity and neuronal damage.^[60]^ However, this process undergoes alterations with aging, because these cells show a reduced capacity for movement and phagocytosis but increase the secretion of proinflammatory cytokines.^[71]^ Consequently, their protective function is lost, triggering neuroinflammation.^[72]^

Neuroinflammation underlies the pathogenesis of neurodegenerative diseases (AD, PD, Huntington's disease (HD), amyotrophic lateral sclerosis (ALS)), in which certain toxic substances (β‐amyloid peptides, α‐synuclein, or dead neurons) activate microglia towards a cytotoxic phenotype M1, which promotes both neurodegeneration and neuroinflammation.[^73^, ^74^, ^75^] This close connection between neurodegeneration and neuroinflammation and the possibility of promoting the polarization of microglia towards an anti‐inflammatory phenotype (M2) is the starting point in the search for new therapeutic approaches that can slow down the progression of the disease.[^73^, ^74^]

## 4 The Role of Endocannabinoid System in Inflammaging

Among the different physiological systems connected to microglial activity and the regulation of cellular and molecular changes in adulthood, the endocannabinoid system (ECS) is worthy of note.[^75^, ^76^, ^77^] The ECS is a ubiquitous neuromodulatory system in our body. It is involved in many physiological functions, e. g., motor coordination, memory, learning, metabolism, food intake, neuronal plasticity, endocrine function, immune response, among others.^[78]^ The ECS primarily includes cannabinoid receptors, endogenous cannabinoids (endocannabinoids) 2‐arachidonoylglycerol (2‐AG) and anandamide (AEA), as well as the enzymes responsible for their synthesis, transport and degradation. Among the cannabinoid receptors, the most abundant is the cannabinoid receptor subtype 1 (CB1R), followed by subtype 2 (CB2R). Transient potential change channel (TRPV) receptors and peroxisome proliferator‐activated receptors (PPAR) are also targets of cannabinoids.^[79]^ Phytocannabinoids, or exogenous cannabinoids (cannabidiol (CBD), tetrahydrocannabinol (THC)), bind to all these receptors producing their biological effects. To date, thanks to its involvement in pathophysiological processes, the ECS is a relevant research topic,^[79]^ particularly focused on the study of CB1 and CB2 receptors. Both receptors are G protein‐coupled receptors (GPCRs); in particular they are coupled to inhibitory G proteins (G_I/O_ proteins),^[80]^ and are characterized by 7‐transmembrane domains, with the N‐terminal end in the extracellular environment and the C‐terminal part inside. The activation of CB1R and CB2R leads to a reduction of adenylate cyclase with reduction of cAMP and activation of mitogen‐activated protein kinases (MAPK).^[81]^ These receptors mediate the effects of endogenous ligands, phytocannabinoids and synthetic molecules. These two receptors share 44 % homology in their amino acidic sequence and differ in the effects mediated by their activation,[^82^, ^83^] also connected to their different distribution in the body.[^83^, ^84^]

CB1R is among the most abundant GPCRs in the CNS.^[85]^ It is abundant in several regions: cerebral cortex, where it is responsible for the ability to make decisions and maintaining the attention threshold; cerebellum and basal ganglia where it manages coordination and motor activity; hippocampus and amygdala where it plays a role in learning, memory and emotion management; hypothalamus where it regulates food intake and appetite; spinal cord where it modulates pain.[^78^, ^85^, ^86^, ^87^, ^88^, ^89^] CB1R is particularly abundant at the presynaptic terminal level where it is responsible for retrograde signaling of endocannabinoids. Thus, it contributes to a feedback inhibition system in the ECS.[^85^, ^86^, ^90^]

The CB2R receptor has a highly diverse anatomical distribution and is involved in the regulation of different physiological functions. It was initially identified at the peripheral level in organs and cells of the immune system, such as thymus, spleen, bone marrow, tonsils, leukocytes, macrophages, T‐lymphocytes, B‐lymphocytes, natural killer cells, monocytes, and mast cells.^[85]^ Recently, the expression of CB2R has been demonstrated in the brain, where under physiological functions it plays a role in neurological functions, such as drug addiction, nociception and neuroinflammation.^[86]^ The anatomical distribution of CB2R in peripheral tissues is remarkable, since it is found in liver, adipose tissue, bones, stomach, intestine, heart and reproductive system.[^86^, ^91^, ^92^] Several studies proved a close correlation between CB2R expression and activated microglia. Under physiological conditions, microglia do not express this receptor, which is instead overexpressed and upregulated in pathological conditions.[^91^, ^93^] For example, overexpression of CB2R has been detected in senile plaques of postmortem brain tissues of patients with AD,[^94^, ^95^, ^96^, ^97^] PD,[^98^, ^99^, ^100^, ^101^] HD[^102^, ^103^] and ALS.[^104^, ^105^]

Although the presence of CB2R in the animal brain under physiological conditions has been known for some time,[^91^, ^106^] only in the last decade, its presence has been found in the human CNS (midbrain,[^107^, ^108^, ^109^] hippocampus,[^107^, ^110^, ^111^, ^112^] brainstem,[^113^, ^114^] cerebellum,[^110^, ^115^] etc). However, the role of CB2R is mainly connected to the modulation of the immune response.[^92^, ^116^, ^117^, ^118^]

**Figure 3:**
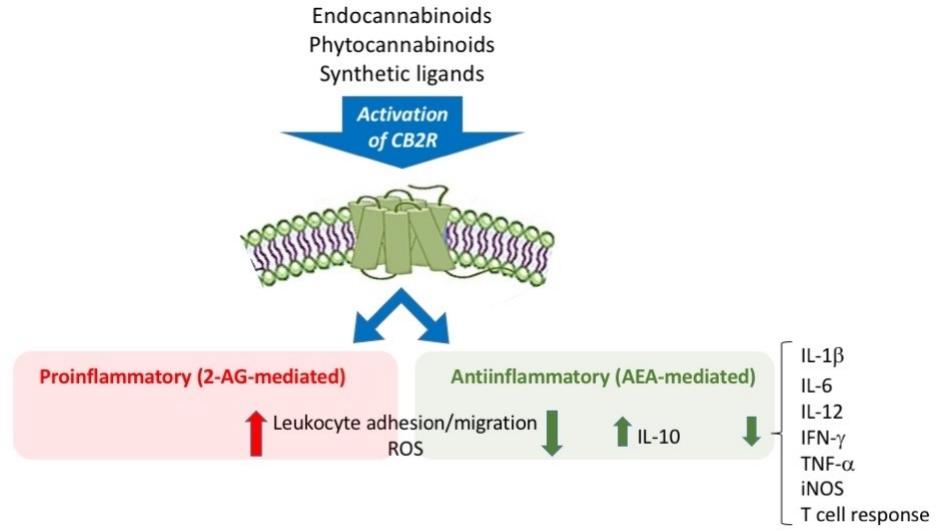
Effects of CB2R activation mediated by endogenous and exogenous ligands (abbreviations in the text).

AEA, 2‐AG as endogenous ligands, THC and CBD as exogenous ligands activate the CB2R by regulating (directly or indirectly) the immune response,^[116]^ i. e., the production of cytokines and chemokines. These substances are signals produced by immune cells capable of binding to and stimulating other immune cells, determining a pro‐ or anti‐inflammatory response (Figure 3).[^116^, ^117^, ^118^] Among the proinflammatory events (purely mediated by 2‐AG) the promotion of leukocyte migration and adhesion is reported, while among the anti‐inflammatory effects (AEA mediated) there are the increase in cytokines with anti‐inflammatory action (IL‐10) and the reduction of proinflammatory cytokines (IL‐6, IL12, IFN‐γ, TNF‐α).^[117]^ Exogenous ligands (phytocannabinoids or synthetic agonists) that stimulate CB2R have an anti‐inflammatory effect since they reduce proinflammatory cytokines and ROS, inhibit leukocyte migration and increase cytokine release.^[118]^ The immunomodulatory action induced by CB2R, as well as the absence of a psychotropic action connected to its action,[^119^, ^120^] make CB2R a therapeutic (but also diagnostic) target of great interest for the treatment of neuroinflammatory pathologies.

### 4.1 Exogenous Ligands that Activate CB2R: An Anti‐Inflammatory Role

ECS has emerged as a key regulator of inflammation and immune response, with the CB2R playing a central role in such processes. CB2R agonists have been the subject of growing interest in medical research due to their ability to modulate inflammation in different pathological contexts. These compounds, of both natural and synthetic origin, have demonstrated potential anti‐inflammatory properties, providing promising therapeutic opportunities for chronic inflammatory disorders and acute inflammatory conditions. CB2R agonists perform their anti‐inflammatory role mainly through the activation of the CB2R, which is present mainly on cells of the immune system, including macrophages, lymphocytes and dendritic cells, but especially microglia. Once activated, the CB2R can modulate several intracellular signaling pathways that influence inflammation. This may include reducing the production of pro‐inflammatory cytokines, inhibiting leukocyte migration to the inflammatory site, promoting apoptosis of activated immune cells, and reducing the production of oxygen free radicals and ROS. Furthermore, CB2R activation can also modulate the immune response in the context of autoimmune diseases, reducing the activity of inflammatory T cells and promoting the differentiation of regulatory T cells. Overall, these mechanisms contribute to the anti‐inflammatory effect of CB2R agonists.[^121^, ^122^, ^123^, ^124^, ^125^, ^126^, ^127^]

Several studies showed an age‐related decrease in endocannabinoid tone due to lowering the amount of 2‐AG and AEA in the brain.[^128^, ^129^, ^130^, ^131^] This event leads to a cognitive decline suggesting the involvement of the ECS in brain aging and in inflammation connected to microglial activity.^[132]^

With advanced age there is a significant alteration in the functionality of the organism. When dysregulation affects the immune system, it triggers the excessive chronic and low‐grade inflammatory response, connected to senescence. In this scenario, phytocannabinoids can be placed for their immunomodulatory functions on the receptors of the ECS system, towards CB2R. CBD and cannabichromene (CBC) for example could reduce the negative effects of inflammaging by shifting the balance towards healthier, non‐inflammatory aging.^[133]^

CBD, a powerful anti‐inflammatory agent, is safe and does not induce psychotropic effects; thus it could be a potential candidate for reversing the signs of inflammaging and refuting inflammation. CBD stimulates the CB2R in different ways, including increasing the activity of the receptor itself or influencing other receptors or pathways that interact with CB2R. When CBD binds to the CB2R, it can modulate the inflammatory response in several ways: (i) reducing production of inflammatory cytokines (CBD can decrease the production of pro‐inflammatory cytokines, thus reducing inflammation); (ii) inhibiting the secretion of inflammatory factors (CBD can block the secretion of inflammatory chemicals by immune cells, helping to reduce inflammation); (iii) modulating the macrophage activity (CBD can influence the activity of macrophages, cells of the immune system involved in inflammation, thus helping to reduce its intensity); (iv) reducing immune cell migration (CBD can limit the migration of inflammatory immune cells to sites of inflammation, thereby reducing the inflammatory response).[^127^, ^134^]

CBC, a phytocannabinoid with a mechanism of action not fully understood yet, may exert its anti‐inflammatory actions through interaction with the CB2R. Its binding to CB2R can lead to a reduction of pro‐inflammatory cytokines by microglial cells. Furthermore, modulation of CB2R can lead to a reduction in the intensity of the inflammatory response.[^135^, ^136^, ^137^]

Synthetic CB2R agonists have become the subject of increasing interest in pharmacological research due to their potential role as anti‐inflammatory agents. By acting on CB2R, synthetic agonists can selectively modulate the inflammatory response, offering a promising therapeutic strategy for a wide range of inflammatory disorders. These compounds, designed to mimic or enhance the endogenous activity of cannabinoids, can act through several mechanisms to reduce inflammation, including suppression of inflammatory cytokine production, inhibition of immune cell migration, and modulation of activity of immune cells.

**Figure 4:**
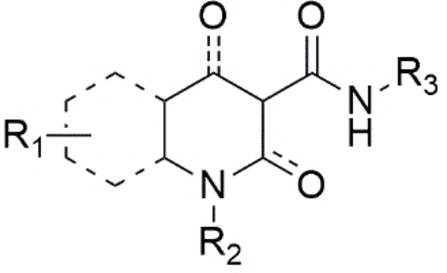
CB2R pharmacophore (adapted from ref. [126]).

To guarantee the agonist effect towards the CB2R receptor, different molecular scaffolds carrying different pharmacophore moieties have been developed, including dibenzopyran, thiophene, imidazopyrazine, oxoquinoline, quinolinedione, indole/indazole, imidazopyridine, benzimidazole and purine.^[126]^ Based on these structures it is possible to identify some fundamental requirements for a common pharmacophore (Figure 4), particularly a mono‐ or bicyclic heterocycle (N, S, O), and an OH or C=O in position 2 or 4 of the scaffold to warrant the formation of hydrogen bonds. The substitution on the heterocycle (R1) must be carefully evaluated because some groups could shift the activity from CB2R to CB1R.^[126]^ The scaffold can have a linear alkyl chain (from 4 to 6 carbon atoms), aromatic or cyclic alkyl groups or even aliphatic cycles with heteroatoms (R2). The CB2/CB1 receptor selectivity is allowed by the presence of the carboxamide bearing bulky groups (aromatic or aliphatic, R3) which also guarantees the activation of CB2R. Aromatic groups interact with specific amino acids located in a deep hydrophobic pocket of CB2R.^[126]^

**Figure 5:**
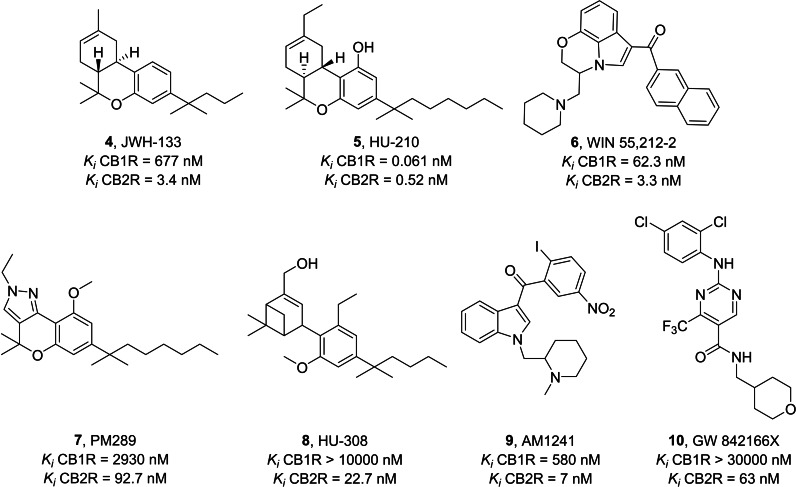
Chemical structures of CB2R agonists.

Among the CB2R agonists, dibenzopyran derivatives are analogues of THC, the natural exogenous ligand obtained from Cannabis plant.^[94]^ JWH‐133 **4** (Figure 5) has strong potential to reduce inflammation caused by amyloid beta proteins involved in AD and plays an essential role in neuronal plasticity under conditions of neuroinflammation and neurodegeneration.^[95]^ Its anti‐inflammatory action is due to the stimulation of the CB2R which blocks the production of proinflammatory biomarkers of the M1 state such as TNF, IL‐6, inducible nitric oxide synthase (iNOS), IL‐1β and other chemokines. Neurotoxicity is reduced by activation of the anti‐inflammatory M2 state resulting in the production of pro‐resolving IL‐10 and arginase‐1.^[138]^ HU‐210 dibenzopyran analogue **5** ^[139]^ and aminoalkylindole derivative WIN55,212‐2 **6** ^[98]^ (Figure 5) are also connected to neuroinflammation in AD, however they are not selective CB2R agonists.[^97^, ^98^, ^99^] PM289 **7** (Figure 5) is a novel pyrazole CB2R agonist. This molecule acts by blocking TNF‐α at the brain level, having a protective role in neuroinflammation.[^100^, ^101^, ^140^]

In Figure 5, the synthetic cannabinoid HU‐308 **8** is a specific CB2R agonist with an effect on systemic inflammation that works by stimulating the production of anti‐inflammatory cytokines.^[102]^ **8** also has an antinociceptive effect, thanks to the inhibition of the proinflammatory M1 state.^[119]^ The agonist AM1241 **9** belongs to the aminoalkylindole family that acts as a potent and selective agonist for the cannabinoid CB2 receptor.[^104^, ^141^] This compound has pain‐relieving but also anti‐inflammatory effects.^[106]^ CB2R activation by **9** reduces the expression of TNF‐α, IL‐1β, and IL‐6.^[107]^ The new chemical structure GW842166X **10**, based on a pyrimidine nucleus (Figure 5), is very promising for the development of CB2R agonists with neuroinflammatory action. It showed high potential for the treatment of inflammatory and neuropathic pain.[^108^, ^142^]

## 5 Formyl Peptide Receptor 2: a new Target in Inflammaging

**Figure 6:**
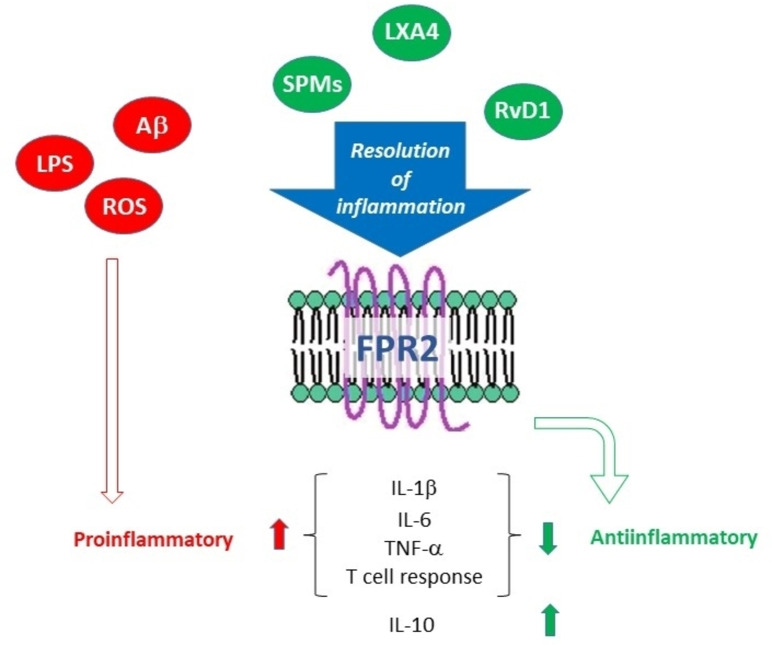
Role of FPR2 receptor in resolution of inflammation (abbreviations in the text).

Formyl peptide receptors 1 and 2 (FPR1, FPR2) are G‐protein coupled receptors expressed in cells from different lineages, including immune cells (neutrophils, macrophages, glial cells and astrocytes).^[143]^ FPR2 plays a crucial role in regulating inflammatory and immune processes, with a particular emphasis on resolution of inflammation (RoI), the physiological process in which the inflammatory response is terminated while simultaneously morphology and functions of the affected tissues are restored. For decades, RoI has been considered a passive event during which inflammation simply fades away, while instead it is a finely regulated process that requires the synthesis of a plethora of mediators, called Specialized Pro‐resolving Mediators (SPMs), that includes lipids and proteins.[^144^, ^145^, ^146^] A piece of evidence highlighted that abnormalities in the RoI are related to chronic inflammation‐based diseases both in periphery and CNS.^[147]^ FPR2 plays a pivotal role in promoting RoI because several SPMs, such as lipoxin A4 (LXA4), resolvin D1 (RvD1) and annexin A1, exert their effects through the interaction with FPR2 (Figure 6).^[148]^

Although the role of FPR2 in inflammaging has been poorly explored, several studies have reported the role of LXA4 and SPMs, in general, in age‐related diseases characterized by chronic inflammation. It is well known that LXA4 and the other SPMs exert various biological actions, including inhibition of chemotaxis, adhesion to/transmigration across endothelial and epithelial cells, and blockade of TNFα secretion from human T cells. It has been hypothesized that aging may disrupt the balance between the pro‐ and anti‐inflammatory mediators that can exacerbate the inflammatory status in elder people, which in turn can favor the onset of age‐related diseases. Moreover, it has been reported that the genetic suppression of 5‐lipoxigenase, the enzyme catalyzing the biosynthesis of LXA4, promotes learning impairment in mice. Conversely, the administration of LXA4 reduces cytokine production and memory loss induced by inflammation in mice, thus suggesting that restoring RoI may result beneficial on chronic inflammation. Furthermore, a correlation of LXA4 has been observed with brain‐derived neurotrophic factor (BDNF) and amyloid‐beta (Aβ) protein accumulation in AD brain.[^149^, ^150^]

Considering that LXA4 serves as endogenous FPR2 ligand, modulating the functional activity of such receptor akin to LXA4 could open a way for treating inflammation in such pathologies. Therefore, in our perspective, targeting FPR2 to stimulate RoI could prove to be an intriguing approach to treat inflammaging. Chronic inflammation, a distinct feature of inflammaging, can potentially be mitigated by actively encouraging the affected microenvironment to release anti‐inflammatory and pro‐resolving cytokines through FPR2 activation.

In recent decades, many efforts have been dedicated by medicinal chemists to developing potent and selective FPR2 agonists. According to the CHEMBL database, from the early 2000s to the present, about one thousand compounds targeting FPR2 have been reported. Here we highlight the most promising small molecules, showing interesting activities in age‐related diseases.

### 5.1 FPR2 Agonists

**Figure 7:**
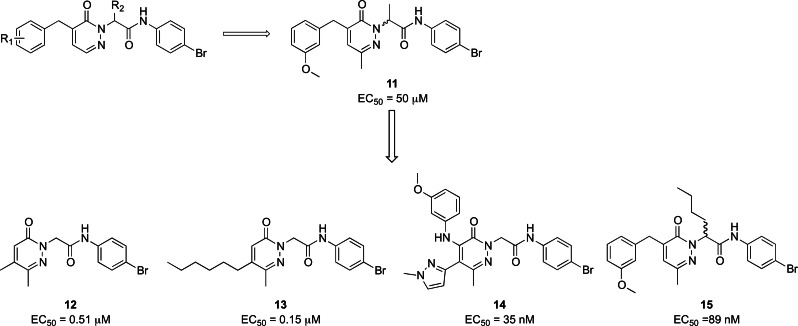
Development of pyridazinone derivatives.

Cilibrizzi et al. developed the FPR2 agonist **11**, featuring a central core containing a pyridazin‐3(2*H*)‐one (Figure 7).^[151]^ The activity of this compound was evaluated based on its ability to induce changes in intracellular calcium mobilization in HL‐60 cells expressing FPR1 along with FPR2 and FPR3, measured in terms of EC_50_. The racemic mixture exhibited an EC_50_ towards FPR2 of 50 μM, whereas in a subsequent paper one of the isolated enantiomers demonstrated an EC_50_ of 10 μM.^[152]^ This compound was developed starting from structurally related FPR1 agonists through structure‐activity relationship (SAR) studies, involving manipulation of the methoxy substituent on the left‐hand side of the molecule and introduction of a methyl group on the right‐hand side, particularly in the acetamide portion. Later, further modifications to compound **11** led to the identification of more potent pyridazin‐3(2*H*)‐one derivatives having EC_50_ values in nanomolar range. These modifications, for example, simplified the left‐hand side by removing the benzyl group and inserting an alkyl chain (**12** and **13**, Figure 7), or altering this portion by incorporating an aryl amine moiety and a 1‐methylpyrazole (**14**, Figure 7).[^153^, ^154^] Another chemical manipulation of **11**, namely the introduction of a *n*‐butyl chain on the stereocenter, lead to a pair of enantiomers **15** (Figure 7), one of which showed a high selectivity and potency toward FPR2, with an EC_50_ of 89 nM.^[155]^

Compound **11** showed cardioprotective activity in myocardial ischemia‐reperfusion injury in mice, vasoprotective activity in streptozotocin‐induced diabetic mice, and vasodilator and anti‐inflammatory activities in mouse precision‐cut lung slices.[^156^, ^157^, ^158^]

Pyridazinone derivatives proved to be themselves intriguing candidates for attenuating inflammaging. They exhibited activity against common pathologies observed in older adults, particularly by exerting anti‐inflammatory effects. This anti‐inflammatory activity is crucial in mitigating inflammaging, making pyridazinone derivatives particularly promising for therapeutic intervention.

**Figure 8:**
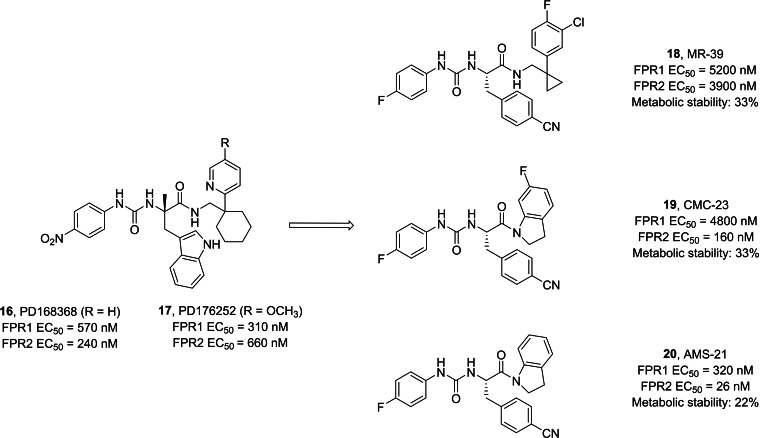
Ureidopropanamides with FPR1/2 activity. Metabolic stability is expressed as % of recovery of parent compound after 30 min incubation with rat microsomes.

In a previous screening, two compounds, PD168368 **16** and PD176252 **17** (Figure 8), initially identified as antagonists of bombesin receptor 2, were unexpectedly found to exhibit characteristics of mixed FPR1 and FPR2 agonists. However, despite their promising activity, these compounds displayed significantly low metabolic stability.[^148^, ^159^] Subsequently, a medicinal chemistry endeavor focused on this molecular scaffold led to the synthesis of a series of phenylureidic derivatives (Figure 8), commonly known as ureidopropanamide derivatives. These compounds were optimized from bombesin antagonists through various structural modifications. The latter included the presence of a fluorine atom in *para* in the phenylureidic moiety on the left‐hand side of the molecule, the removal of the methyl group from the C asymmetric center, and manipulation of the amino acidic central core, particularly the lateral chain of the amino acid, namely switching from tryptophan to 4‐CN phenylalanine. Furthermore, significant alterations have been made on the right‐hand side of the molecule, which included the introduction of various moieties, such as a phenylcyclopropylmethyl moiety, an indoline, and a 6‐fluoroindoline, which are integral components of the most active and stable compounds. All these modifications aimed at achieving the optimal balance between enhancing the pharmacodynamic profile and ensuring favorable pharmacokinetic profiles to develop candidates for in vivo studies. Remarkably, all these compounds exhibited activity at FPR2 within the range of 3900 nM to 24 nM, and they were found to be the most stable compounds, as evidenced by their half‐life after incubation with rat microsomes.[^148^, ^160^, ^161^, ^162^, ^163^]

Ureidopropanamides proved to be effective agents with pro‐resolving and neuroprotective properties across various models. They consistently regulate the inflammatory profile of affected tissues, particularly by attenuating inflammation and, notably, promoting pro‐resolving effects.^[148]^ Compound MR‐39 **18** showed anti‐inflammatory, pro‐resolving, and neuroprotective effects in neuroinflammatory processes. These effects were observed in organotypic hippocampal cultures (OHCs) exposed to bacterial endotoxin (LPS), as well as in LPS‐exposed microglia cells.[^164^, ^165^] Additionally, it showed potential in improving the inflammatory profile in ex vivo and in vivo Aβ(1‐42)‐induced neuroinflammation in mouse models of AD. Specifically, compound **18** led to a reduction in the release of pro‐inflammatory cytokines while enhancing the release of anti‐inflammatory and pro‐resolving cytokines such as IL‐10.^[166]^ Specifically, pretreatment with **18** abolished some of the LPS‐induced changes in the expression of genes related to microglia polarization towards M1/M2 phenotypes (including IL‐1β, IL‐6, Arg1, IL‐4, Cd74, Fizz, and Cx3cr1). Additionally, **18** attenuated the LPS‐ or Aβ(1‐42)‐induced increase in the levels of inflammasome NLRP3 and caspase‐1 proteins through the interaction with FPR2, as such effects were abolished by pre‐treatment with the selective FPR2 antagonist WRW4 or were absent in organotypic cultures from FPR2 knock‐out mice.[^164^, ^165^, ^166^] The anti‐inflammatory and pro‐resolving properties of the ureidopropanamide‐based FPR2 agonists has been further confirmed. In fact, compound CMC‐23 **19** showed anti‐inflammatory and pro‐resolving effects in LPS‐stimulated OHCs and this effect was mediated through the modulation of the STAT3/SOCS3 signaling pathway.^[167]^ In addition, it was also demonstrated that pro‐resolving effects of ureidopropanamide‐based FPR2 agonists were mediated through the involvement of FPR2 expressed by microglial cells, as these effects were not observed in microglia‐depleted hippocampal organotypic cultures.^[168]^ Moreover, promoting RoI by targeting FPR2 can modulate the hippocampal pro‐inflammatory profile, neuronal plasticity, and social behavior in two validated animal models of autism spectrum disorder (ASD), i. e., the BTBR mouse strain and mice prenatally exposed to valproic acid. It notably reduced several inflammatory markers, restored the low expression of LXA4, and modulated FPR2 expression in hippocampal tissues.^[169]^ Even biochemical properties of compounds **19** and AMS‐21 **20** were evaluated deeper, showing pro‐resolving and neuroprotective properties in OHCs.[^161^, ^168^]

Neurodegenerative diseases are prevalent conditions in older adults, characterized by chronic inflammation alongside other features. The above discussed studies strongly support the notion that targeting FPR2 can attenuate chronic inflammatory responses in CNS disorders, thus offering a valuable therapeutic option for combating inflammaging.

**Figure 9:**
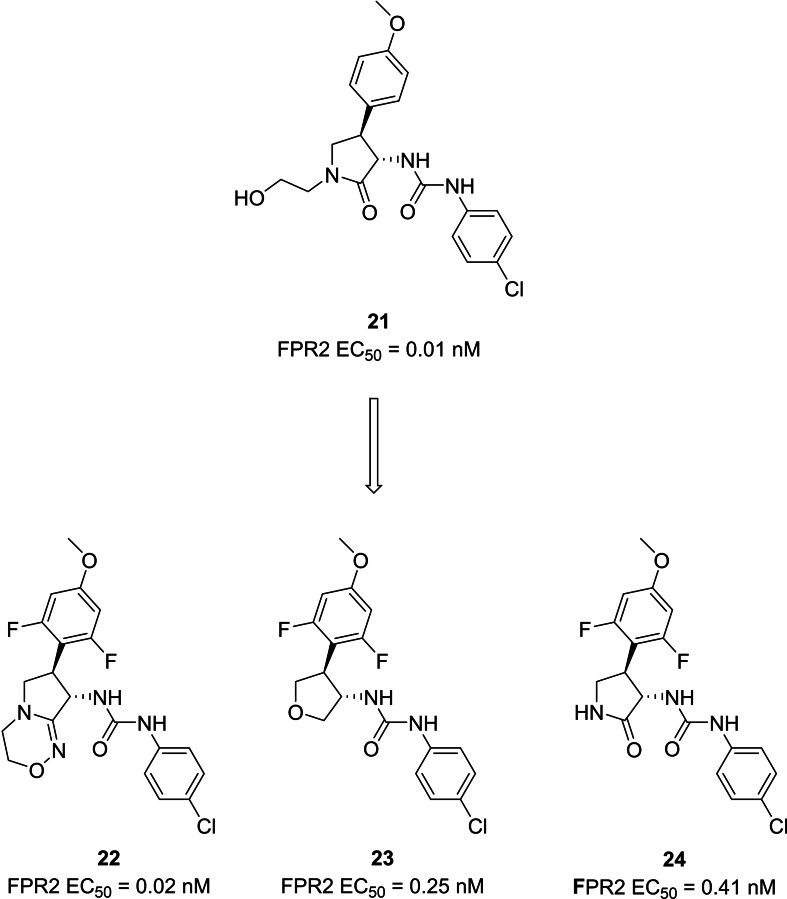
Patented phenylurea derivatives with FPR2 agonist activity.

Both academic research and pharmaceutical companies are actively contributing to the identification of FPR2 agonists. For example, several patents claimed the identification of FPR2 agonists endowed a phenylurea scaffold, a pyrrolidone or tetrahydrofuran‐based moieties (Figure 9). These compounds proved to be able to activate FPR2 in the nanomolar or picomolar range. Starting from the identification of the first pyrrolidone‐based compound **21**,^[170]^ the optimization brought to the synthesis of very potent derivatives with either a fused bicycle (**22**) instead of the pyrrolidone, or a tetrahydrofuran (**23**).^[171]^ Further modifications led to compound **24**, a pyrrolidone‐based derivative, which reached phase 1 stage of clinical trials, as a candidate for cardiovascular diseases.[^172^, ^173^]

**Figure 10:**
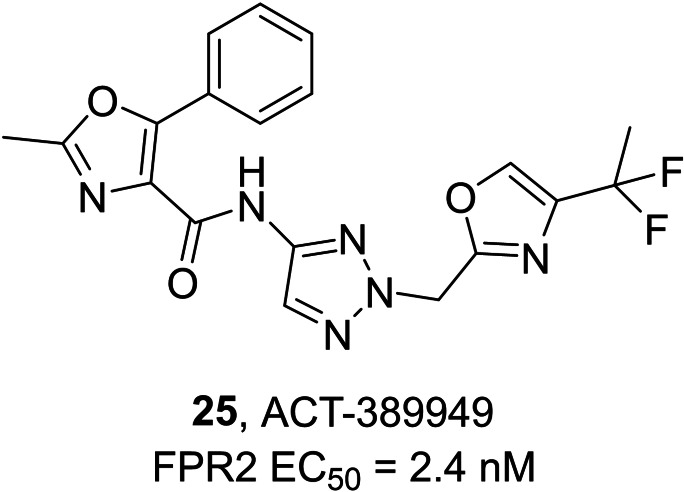
Patented derivative with FPR2 agonist activity.

Actelion Pharmaceuticals developed another series of compounds acting as FPR2 agonists with aminotriazole, aminopyrazole or aminoimidazole scaffolds, leading to the development of compound ACT‐389949 **25** (Figure 10), which has been evaluated in phase 1 clinical trial for inflammatory diseases.^[174]^

## 6 Oxidative Stress and Immunosenescence

At balanced basal levels, ROS behave as signaling molecules and are involved in the regulation of numerous physiological events, such as cell growth and signaling, metabolism, synthesis of biological molecules, post‐translational processing of proteins and the immune response.[^175^, ^176^] In contrast, a sustained/excessive amount of ROS causes damage to proteins, DNA, lipids and lipid membranes also altering cellular fluidity and permeability.

The term ‘oxidative stress’, first coined in 1985,^[177]^ relates to the redox imbalance and its cytotoxic consequences. Recently, there has been growing evidence suggesting a strong interplay between oxidative stress, inflammation, and immunosenescence.^[178]^ These conditions pave the way for the risks of ARDs such as degenerative diseases of the CNS.[^179^, ^180^, ^181^]

**Figure 11:**
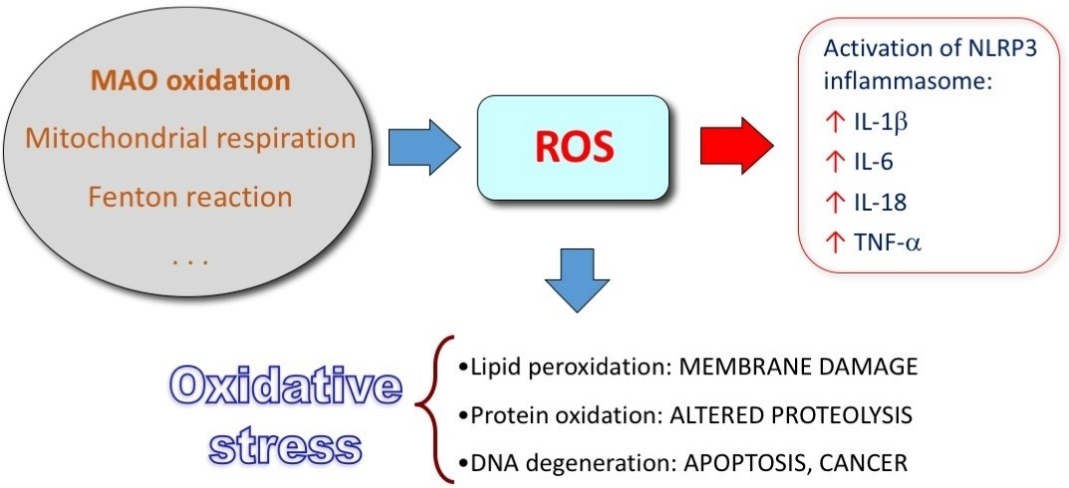
Activation of inflammatory response and oxidative stress by ROS (abbreviations in the text). ROS can be generated by metabolic reactions (MAO oxidation, mitochondrial respiration), chemical reactions (Fenton reaction, i. e., Fe(II)‐dependent generation of hydroxyl radicals from hydrogen peroxide) and exogenous sources of ROS.

Increased amount of ROS activate directly the NLRP3 inflammasome, that is a multiprotein complex in the cytoplasm, which generates active caspase 1, converting pro‐IL1β and pro‐IL‐18 into active cytokines (Figure 11).[^182^, ^183^] Additionally, the products of oxidative damage can trigger an inflammatory response by means of TLRs. TLRs constitute a family of ubiquitous receptors that detect the presence of infection and induce innate immune responses. Via the MyD88 adaptor, the activation of TLR8 and TLR4 pathways initiates an inflammatory response whose key mediators are IL‐1, IL‐6 and TNF‐α.[^184^, ^185^, ^186^] Therefore, granulocytes, macrophages and dendritic cells are constantly activated, and ROS are produced, which results in a vicious circle.

Immunosenescence impairs both the adaptive and the innate immune system. It consists in alteration of immune function and a consequent reduced rate of senescent cell clearance.^[187]^ Interestingly, the resulting accumulation of senescent cells further support the inflammation.^[188]^ It is reported that with aging there is an increase of the amount of ROS in immune cell system,^[189]^ as well as a decrease of the level of antioxidant defenses in cytosol and mitochondria.^[190]^ The chronic exposure to oxidative stress over time is the main cause of the changes in immune cell function (among others, e. g., the alteration of endosomal organelles compromising the pathogen processing ability of antigen presenting cells) and the cause of the increase of cells undergoing apoptosis.

**Figure 12:**
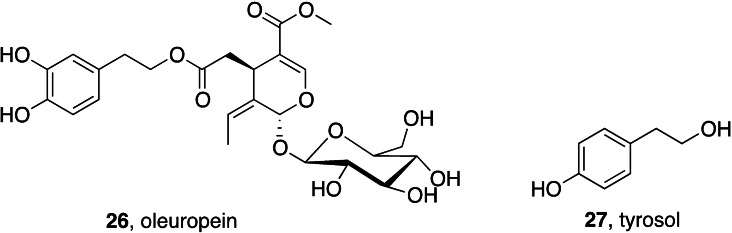
Natural antioxidants for inflammaging.

Natural antioxidants have long been considered privileged nutraceuticals. The dietary intake of antioxidants is assumed to counteract the effects of oxidative stress in triggering and sustaining inflammaging.^[191]^ The efficacy of oleuropein **26** and tyrosol **27** (Figure 12), two antioxidants present in several foods of the Mediterranean diet, in ameliorating inflammaging has been recently reviewed.^[192]^

## 7 Monoamine Oxidase Inhibitors as Neuroprotective Agents

Monoamine oxidase (MAO) activity is viewed as an endogenous source of ROS. MAO is a flavin‐dependent enzyme, located in the inner side of outer mitochondrial membrane. Two MAO isoforms, A and B, have distinct properties regarding their localization^[193]^ and specificity for substrates and inhibitors.^[194]^ They catalyze the oxidative deamination of neurotransmitters and exogenous aryl/alkylamines (serotonin, dopamine, norepinephrine, epinephrine, β‐phenethylamine and other trace amines, such as tyramine and tryptamine) using oxygen as the final electron acceptor and producing the substrate related aldehydes, ammonia or a primary amine and hydrogen peroxide (H_2_O_2_) involved in the oxidative stress process by generating a variety of ROS.^[195]^ The development of selective MAO B inhibitors is useful to avoid the so‐called cheese effect (tyramine‐induced hypertensive crisis) which is linked to peripheral MAO A inhibition. Additionally, non‐selective inhibitors improve the possibility of interaction with other medications and are therefore marked with an FDA black box warning.^[196]^

Some of the known MAO B inhibitors include chalcones, pyrazolines, oxadiazole, chromones, xanthines, coumarins, thiazole, thiazolidine, hydrazones, isatin congeners and propargylamine derivatives. Herein, we show some of the most relevant works focused on the use of MAO B inhibitors in neuroinflammation in the last four years.

Many efforts have been addressed to obtain multifunctional drugs with different combination of targets to harness the synergistic potential of therapeutic targets managing neurodegenerative diseases.

**Figure 13:**
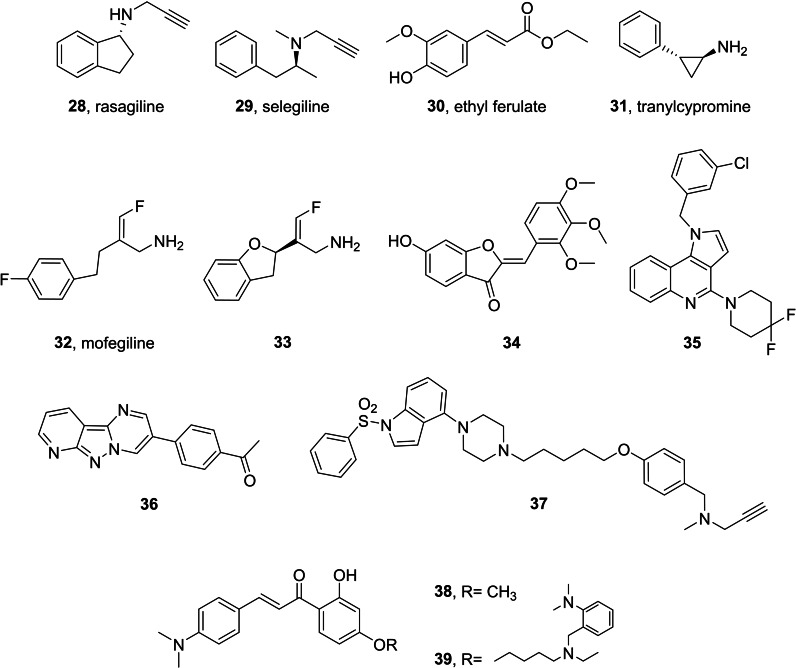
MAO inhibitors with neuroprotective activity.

The group of Viola and Canton^[197]^ evaluated the effect of rasagiline **28** (Figure 13), a clinical‐grade MAO B inhibitor, on inflammation. The study in murine bone marrow‐derived macrophages triggered by LPS/ATP showed that the drug significantly reduced IL‐1β secretion. In vivo, they challenged mice with intraperitoneal LPS injection and analyzed the levels of circulating IL‐1β, TNF‐α, and monocyte chemoattractant protein‐1 (MCP‐1) and they found that rasagiline pretreatment reduced IL‐1β production in response to LPS stimulation and dampened the recruitment of inflammatory cells into the peritoneal cavity.

It has been recently reported that the administration of selegiline **29** (irreversible inhibitor of MAO B, Figure 13) during action‐outcome remapping rescues the neuronal, learning‐, and memory‐related deficits in goal‐directed action control, normalizing striatal cholinergic interneurons and goal‐directed control after inflammation‐induced damage to the neurons in parafascicular thalamus.^[198]^

Ethyl ferulate (**30**, Figure 13) is a natural compound having an anti‐inflammatory effect on LPS‐induced acute lung injury^[199]^ and able to protect neurons from Aβ(1‐42)‐induced oxidative stress in AD models.^[200]^ The molecular target of **30** was unclear but Zou et al.^[201]^ showed that **30** is a MAO B inhibitor, explaining its therapeutic effect in microglia mediated neuroinflammation. Their results indicated that co‐treatment of **30** with rasagiline in LPS‐activated BV2 cells did not show additive effect in MAO activity and LPS‐induced pro‐inflammatory effect, indicating that **30** suppressed microglia‐mediated neuroinflammation in a MAO‐dependent manner.

Another study reported that tranylcypromine **31** (irreversible MAO inhibitor, Figure 13) regulates LPS‐ and Aβ‐mediated neuroinflammatory responses in BV2 microglial cells, wild‐type, and 5XFAD mice.^[202]^

Mofegiline **32** (Figure 13) was under development for Parkinson's disease in the 1990s, reaching phase 2 clinical trials. It is a dual MAO B/semicarbazide‐sensitive amine oxidase (SSAO) inhibitor member of the fluoroallylamine class of amine oxidase inhibitors, a class of irreversible inhibitors.[^203^, ^204^] Using this compound as starting lead, Foot et al.^[205]^ developed the analogue **33** (IC_50_ SSAO=19 nM, MAO B=3 nM) (Figure 13). It showed a good physicochemical profile leading to a reduced risk of negative effects on cell health in vitro, improved selectivity over MAO−A, and excellent drug‐likeness properties as assessed by in vitro ADME assays. It also demonstrated anti‐inflammatory properties in an acute model of neutrophil‐driven inflammation, reducing both infiltrate volume and relative neutrophil numbers. Moreover, in a model of acute inflammation, at 6 mg/kg it reduced the total inflammatory cell volume into the air pouch and led to a >50 % reduction in neutrophil numbers. The hit therapeutic dose (tested at 0.6 mg/kg/day and at 6 mg/kg/day) reduced the severity of the clinical manifestations of the relapse in experimental autoimmune encephalomyelitis (EAE) mouse model,^[206]^ one of the most notable neuroinflammatory disease animal model.

The chromone (benzopyran‐4‐one) scaffold can be easily functionalized by suitable substitution patterns to obtain potent and selective MAO B inhibitors. Hassan et al.^[207]^ have prepared a library of compound starting from the natural product hispidol, a 4’,6‐dihydroxyaurone previously described for its capacity of inhibiting MAO A.^[208]^ The positional scanning made in the work consists in the exploration of mono‐methoxylated ring B regioisomers, different dimethoxylation patterns of ring B, and trimethoxylated ring B and the introduction, in some congeners, of the methoxy group in position 6 of the ring A of the aurone. Within this library, compound **34** (Figure 13) emerged as a reversible, potent (IC_50_=171 nM), and highly selective MAO B inhibitor (SI >583), while tests in BV2 cells showed inhibition of NO and PGE2 production.

Recently, Grychowska et al.^[209]^ have introduced fluorine atoms in 1*H*‐pyrrolo^[3,2‐*c*]^quinoline derivatives capable of inhibiting monoamine oxidase type B (MAO−B) in order to reduce the affinity for ether‐a‐go‐go‐related gene (hERG) channel, which is responsible for the prolongation of the QT interval. In vitro studies reveal that the synthetic compound **35** (Figure 13) is a reversible inhibitor of MAO B (IC_50_=9 nM) with no activity for MAO A isoform, low affinity for the hERG channel, good metabolic stability, favorable safety profile, and brain permeability. Finally, it shows glioprotective effects against DOX in a model of cultured astrocytes and in in vivo evaluation it revers scopolamine‐induced cognitive deficits in the NOR test in rats and displays antidepressant‐like activity in mice.

Accounting on literature reports about tricyclic MAO inhibitors,[^210^, ^211^] Jismy et al.^[212]^ have explored the chemical space of pyrimido^[1,2‐*b*]^indazoles by preparing a small library of (hetero)aryl derivatives. They obtained several reversible and competitive inhibitor of MAO B; among these, analogue **36** (IC_50_=130 nM; Figure 13) protected SH‐SY5Y cells against 6‐hydroxydopamine‐induced cell death.

The approach followed by Canale et al.^[213]^ consisted in linking via alkyl chain of different length the aryloxy fragments derived from reversible and irreversible MAO B inhibitors with a (indol‐4‐yl)‐piperazine fragment that is a well‐established core of serotonin receptor 5‐HT_6_R antagonists. Compound **37** acted as an inverse agonist of 5‐HT_6_R and an irreversible MAO B inhibitor (*K*_i_ 5‐HT_6_R=38 nM, IC_50_ MAO B=154 nM; Figure 13). Compound **37** showed moderate metabolic stability, brain penetration, and no hepatotoxicity. Additionally, it displayed glioprotective properties from 6‐OHDA in C8‐D1 A astrocytes and it also reversed the cognitive decline induced by scopolamine in the NOR test.

Chalcone (1‐phenyl‐2‐benzoylethylene) and its derivatives have been reported to have several activities, namely radical‐scavenging, metal chelation, anti‐inflammatory, AChE inhibition, neuroprotective MAO B inhibition.[^214^, ^215^] Sang et al.^[216]^ have designed and synthesized a series of dimethylamino chalcone‐O‐alkylamine, by fusing multiple pharmacophores assessed for AD treatment. In general, dimethylamino group at 4‐position of chalcone skeleton (Figure 13) exhibited better inhibitory activity than at 4’‐position. Compound **38** was found as the best MAO B inhibitor (IC_50_=212 nM, SI=36.3). Within the studied series, **39** was less active on MAO B (IC_50_=1.0 μM; SI=18.4) but showed the greatest inhibitory activity against self‐induced Aβ aggregation, a remarkable antioxidant activity, and the higher AChE inhibition.

## 8 Concluding Remarks

Inflammaging and immunosenescence are closely linked phenomena that contribute to the decline in aging health. The COVID pandemics have exacerbated the need of addressing these chronic pathologies through pharmacological interventions. The connection between immune frailty and age has been largely evidenced, although only few studies have provided epidemiological studies. Concerning COVID severity in elderly, sex disparity has demonstrated to play a role, mostly because of different influence of sex hormones.^[217]^ Other studies found a relationship between socio‐economic conditions and immunosenescence in a cohort of US people aged >56.^[218]^

Since inflammaging and immunosenescence share common inflammatory pathways and mechanisms, drugs targeting these pathways can potentially address both conditions simultaneously. Both steroidal and non‐steroidal antiinflammatory drugs find clinical use, although their long‐term use is limited by side effects such as gastrointestinal and cardiovascular risks. The therapeutic potential of anticoagulant drugs, described in COVID acute infection,^[219]^ is also being investigated for anti‐inflammatory drugs in post‐COVID syndromes.^[220]^ Immunotherapy has gained increasing attention in this field, particularly the cell‐mediated clearance of senescent cells by senolytic CAR−T cells^[221]^ and the reactivation of senescent immune system exerted by myeloid‐derived suppressor cells (MDSC)^[222]^ and regulatory T (Treg) cells^[223]^ for the rebalancing of inflammaging. Anti‐cytokine therapies targeting specific cytokines, such as IL‐6 or TNF‐alpha, have been proposed to directly modulate inflammatory responses,^[224]^ though their use must be carefully managed to avoid compromising immune functions.

The process of drug discovery from small molecules is benefiting of the new computational tools applying artificial intelligence (AI) techniques to drug design and development, particularly useful into the complex framework of inflammaging/immunosenescence.^[225]^ Designing of new drugs, or repositioning of old ones, may be achieved by predictive AI‐based models, able to select the best activity profile according to different pathological conditions in which inflammaging and immunosenescence are involved.

In this scenario, ‘classical’ small molecules, possibly addressing alternative targets of inflammation, still represent a valid matter of investigation. In this review we described some of these targets, currently investigated also in our laboratories, their role in the biochemical pathways of inflammaging and their potential as therapeutic agents. Of course, besides the potential benefits in counteracting inflammaging and immunosenescence, and in reducing the incidence and severity of age‐related diseases, great attention must be devoted to the assessment of side effects and long‐term safety, and to individual variability in clinical responses. In this light, multitarget agents exerting different pharmacological properties in a single molecular entity can synergistically combine therapeutic benefits, as well as advantages in terms of safer (and faster assessment of) pharmacokinetic profiling.

## Abbreviations

2‐AG: 2‐arachidonoylglycerol
5‐HT6R: serotonin receptor subtype 6
6‐OHDA: 6‐hydroxydopamine
Aβ: beta amyloid protein
AChE: acetylcholinesterase
AD: Alzheimer's disease
AEA: anandamide
ALS: amyotrophic lateral sclerosis
ARD: age‐related diseases
ASD: autism spectrum disorder
BDNF: brain‐derived neurotrophic factor
CB1R: CB2R, cannabinoid receptor subtype 1 and 2
CBC: cannabichromene
CBD: cannabidiol
COVID‐19: COVID, coronavirus disease (2019)
EAE: experimental autoimmune encephalomyelitis
ECS: endocannabinoid system
EF: Ethyl ferulate
FPR1: FPR2, FPR3, formyl peptide 1, 2 and 3 receptor
GPCR: G protein‐coupled receptors
HD: Huntington's disease
hERG: ether‐a‐go‐go‐related gene
IFN: interferon
IGF‐1: insulin‐like growth factor 1
IL: interleukin
iNOS: inducible nitric oxide synthase
LHS: left‐hand side
LPS: lipopolysaccharide
LXA4: lipoxin A4
MAO AB: monoamine oxidase A and B
MCP‐1: monocyte chemoattractant protein‐1
NF‐kB: nuclear factor kappa B
NGF: nerve growth factor
NLRP3: NOD‐like receptor protein 3
NO: nitric oxide
NOR: novel object recognition test
OHC: organotypic hippocampal culture
PD: Parkinson's disease
PPAR: peroxisome proliferator‐activated receptors
RHS: right‐hand side
RoI: resolution of inflammation
ROS: reactive oxygen species
RvD1: resolvin D1
SAR: structure‐activity relationships
SARS‐CoV‐2: severe acute respiratory syndrome coronavirus 2
SPM: specialized pro‐resolving mediator
SSAO: semicarbazide‐sensitive amine oxidase
TGF‐β: transforming growth factor beta
THC: tetrahydrocannabinol
TLR: toll‐like receptor
TNF‐α: tumor necrosis factor α
TRPV: transient potential change channel receptors

## Conflict of Interests

The authors declare no conflict of interest.

## Biographical Information

*Marco Catto is full professor of medicinal chemistry at the Department of Pharmacy‐Pharmaceutical Sciences of the University of Bari. He obtained his PhD in Medicinal Chemistry in 2003 at the same University, where he became assistant professor in 2006. He has been visiting researcher at the universities of Heidelberg (D), Geneva (CH), and Caen (F). His research is dedicated to the synthesis and the biological and physicochemical characterization of multitarget ligands of neurodegenerative‐ and cancer‐related targets, particularly MAO/AChE inhibitors, disruptors of beta‐amyloid and tau aggregation, nucleic acid binders*.

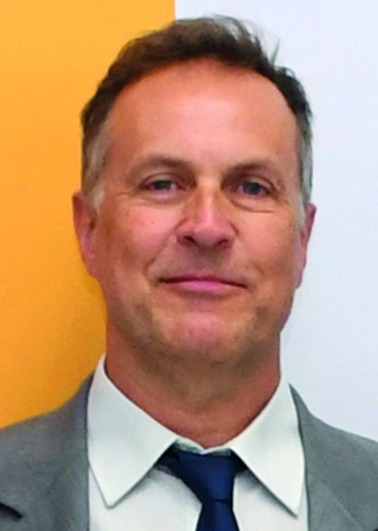

## Data Availability

Data sharing is not applicable to this article as no new data were created or analyzed in this study.
